# Biophysics Role and Biomimetic Culture Systems of ECM Stiffness in Cancer EMT

**DOI:** 10.1002/gch2.202100094

**Published:** 2022-03-20

**Authors:** Hao Tian, Hanhan Shi, Jie Yu, Shengfang Ge, Jing Ruan

**Affiliations:** ^1^ Department of Ophthalmology Shanghai Key Laboratory of Orbital Diseases and Ocular Oncology Ninth People's Hospital Shanghai JiaoTong University School of Medicine Shanghai P. R. China

**Keywords:** biomimetic culture system, epithelial‐mesenchymal transition stiffness, epithelial‐mesenchymal transition, mechanotransduction, tumor microenvironment

## Abstract

Oncological diseases have become the second leading cause of death from noncommunicable diseases worldwide and a major threat to human health. With the continuous progress in cancer research, the mechanical cues from the tumor microenvironment environment (TME) have been found to play an irreplaceable role in the progression of many cancers. As the main extracellular mechanical signal carrier, extracellular matrix (ECM) stiffness may influence cancer progression through biomechanical transduction to modify downstream gene expression, promote epithelial‐mesenchymal transition (EMT), and regulate the stemness of cancer cells. EMT is an important mechanism that induces cancer cell metastasis and is closely influenced by ECM stiffness, either independently or in conjunction with other molecules. In this review, the unique role of ECM stiffness in EMT in different kinds of cancers is first summarized. By continually examining the significance of ECM stiffness in cancer progression, a biomimetic culture system based on 3D manufacturing and novel material technologies is developed to mimic ECM stiffness. The authors then look back on the novel development of the ECM stiffness biomimetic culture systems and finally provide new insights into ECM stiffness in cancer progression which can broaden the fields’ horizons with a view toward developing new cancer diagnosis methods and therapies.

## Graphical abstract

Extracellular matrix (ECM) stiffening is not only a disease consequence but also a contributing factor to disease development. ECM stiffness can independently initiate epithelial‐mesenchymal transition (EMT) or act as a costimulatory or “catalyst” in the tumor cell EMT process. Meanwhile, novel ECM stiffness biomimetic culture systems provide more suitable choices for the biophysical study of ECM.

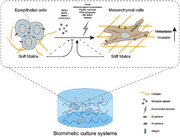

## 1 Introduction

The number of global deaths from noncommunicable diseases has been rising steadily, driven by aging and population growth, as oncological diseases rank second only to cardiovascular diseases in this context.^[^ ^1^ ^]^ Since the discovery of cancer, researchers have never stopped finding pivotal information in tumor development. Until the 1980s, researchers had focused on tumor‐centric views and ignored the microenvironments of tumors.^[^ ^2^ ^]^ However, due to the limitations of tumor‐centricity and breakthroughs in tumor microenvironment environment (TME) related studies, scientists have turned their eyes to the impact of the TME on cancer progression. The TME is composed of cellular components and extracellular matrix (ECM). Earlier studies focused mainly on the effect of cellular components of the TME such as fibroblasts, immune cells, and endothelial cells,^[^ ^3^ ^]^ whereas now the widely accepted view is that the ECM, the dominant component, plays an important role in tumor development, progression, and metastasis.^[^ ^4^ ^]^

The ECM, as the skeleton of tissues and organs, is composed of various proteins such as collagen, proteoglycans (PGs), and glycoproteins. These proteins interconnect with other ECM molecules to construct a complex 3D matrix network, some of which can interact directly with tumor cells through cell membrane surface receptors to modify cellular processes.^[^ ^5^ ^]^ In addition to biochemical properties, ECM also possesses biophysical parameters such as topography, molecular density, stiffness, and tension.^[^ ^4^ ^]^

Mechanical force is a signal vehicle by which cells sense the disturbance in their environment. Matrix stiffness along with elastic energy, hydrostatic pressure, shear force, and local forces from neighboring cells are the major mechanical environments faced by cells, which could induce cells to respond and react in a specific way and play a crucial role in tissue homeostasis.^[^ ^6^ ^]^ While during tumorigenesis, which is usually accompanied by increased tissue stiffness, the mechanical balance is disturbed.^[^ ^7^ ^]^ Matrix stiffness, as a vital mechanical effector, could promote tumor occurrence, proliferation, invasion, metastasis, and drug response through mechanical transduction pathways and physical compression of tumor blood vessels.^[^ ^8^ ^]^ Recently, many studies found that an elevation in ECM stiffness could induce epithelial‐mesenchymal transformation (EMT) of tumor cells independently or with other costimulators.^[^ ^9^ ^]^ EMT in cancers is related to cancer cell invasion, metastasis, stemness, and chemoresistance,^[^ ^10^ ^]^ that have attracted increasing attention in recent years.^[^ ^11^ ^]^ Considering the remarkable correlation between EMT and poor prognosis in cancer, the effect of ECM stiffness on tumor EMT should be thoroughly investigated.

Inescapably, traditional cell cultures based on plates lack controllable physical properties. To better understand the unique effects of matrix stiffness in cancer progression, researchers have applied different materials to simulate ECM, such as natural matrix proteins and polymers, for in vitro experiments.^[^ ^12^ ^]^ Different materials have various characteristics, but in practical applications, original materials cannot perfectly meet the requirements due to their shortcomings. To better simulate the in vivo environment, researchers have been looking for new biomaterials or improved methods for preparing culture systems.

For the sake of better understanding the interaction mechanism of ECM stiffness in cancer progression and to provide a reference for subsequent studies on matrix stiffness, we summarized herein the findings of ECM stiffness in cancer EMT as well as the development of in vitro culture systems in recent years mimicking ECM stiffness, expecting to promote the studies of the potential effects of ECM stiffness on cancer progression and further provide new solutions for oncology therapy.

## 2 ECM Stiffness Formation and Mechanism Affecting Cancer

### 2.1 Stiffness from Physiology to Pathology

Stiffness, representing the extent of anti‐deformation when a material resists deformation under stress, is used to describe whether the material is pliable (soft) or rigid (hard).^[^ ^8b^ ^]^ In biology, stiffness is one of the mechanical properties of the matrix. Different tissues have different ECM stiffness under physiological conditions, and the same tissue can present different matrix stiffness under pathological conditions (**Table** **1**). For example, tumors are usually stiffer than normal tissue, breast tumors are 10 times harder than healthy breast tissue.^[^ ^13^ ^]^ Thus, in the clinic, the easiest way to diagnose a tumor clinically is through palpation.

**Table 1:** Elastic moduli of different tissues under physiological and pathological status

Tissue	Physical stiffness	Pathological stiffness	Ref.
Lung	150–200 Pa	15 kPa (Fibrosis)	^[^ ^149^ ^]^
Brain	50–450 Pa	7–26.7 kPa (Glioblastoma)	^[^ ^142^, ^144^, ^149^, ^150^ ^]^
Breast	800 Pa	5–10 kPa (Cancer)	^[^ ^33^, ^149^, ^151^ ^]^
Gastric	≈0.5–1 kPa	≈7 kPa (Cancer)	^[^ ^152^ ^]^
Pancreas	1–2.9 kPa	3.7 kPa (Cancer)	^[^ ^153^ ^]^
Liver	2.1 kPa	>6 kPa (Cirrhosis)	^[^ ^154^ ^]^
Skin	80–160 MPa	/	^[^ ^155^ ^]^
Bone	2–5 GPa	/	^[^ ^149a^ ^]^

The ECM is a highly ordered meshwork that continuously undergoes controlled remodeling, involving quantitative and qualitative changes. Each organ has a unique, constantly remodeled ECM in the early stages of embryo development, and ECM stiffness is deemed to be a critical factor in embryonic development.^[^ ^14^ ^]^ Once the order is disrupted, dysregulation of ECM can induce several pathological conditions, such as fibrosis and cancer, where matrix stiffening usually occurs.^[^ ^14a^ ^]^ The stiff ECM can reduce the polarity of the tissue, destroy the adhesion, and lead to increased proliferation of tumor cells.^[^ ^15^ ^]^ However, during tumorigenesis, many factors in the tumor environment, such as hypoxia and TGFβ can activate tumor stromal cells, especially cancer‐associated fibroblasts (CAFs), in turn changing the ECM and resulting in excessive deposition of ECM components, crosslinking of collagen, and release of proteolytic enzymes, thus increasing matrix stiffness.^[^ ^16^ ^]^

### 2.2 How the ECM Becomes Stiff

**Figure 1:**
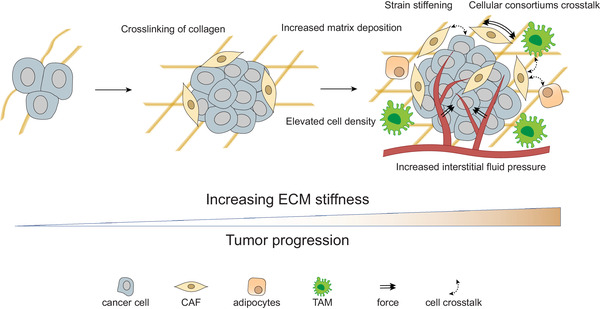
With the tumor progression, the ECM stiffness is generally increasing, mainly due to the increased matrix deposition, the crosslinking of collagen, strain stiffening, elevated cell density, and increased interstitial fluid pressure. Besides, the crosstalking‐between cellular consortiums in tumors facilitated this process.

There were many factors contributing to the stiffening of tumor ECM, including biochemical factors, physical factors, and intercellular interactions (**Figure** **1**).

#### 2.2.1 Increased Matrix Deposition

A major cause of ECM stiffen is the disruption of the balance between deposition and degradation of ECM, leading to an increase in the concentration of matrix proteins in the TME.^[^ ^17^ ^]^ The correlation between some kinds of ECM proteins and matrix stiffness has been revealed. For instance, the colorectal cancer areas that are rich in collagen and increased collagen arrangement are stiffer than normal colon tissues, indicating that an increased quantity of collagen and changes in its structure might play an important role in ECM stiffness.^[^ ^18^ ^]^ The expansion of matrisome glycoproteins and PGs is also found to increase the modulus of tissues significantly through glycosylation and cross‐linking.^[^ ^19^ ^]^

#### 2.2.2 Crosslinking of Collagen

Collagen is the most common matrix scaffold protein that contributes to the tensile strength of tissues, and crosslinking of collagen could increase the ECM resistance by augmenting ECM elastic modulus. Collagen crosslinking is mediated mainly by lysyl oxidase (LOX) and LOX‐like enzymes (LOXLs). LOX and LOXLs are often overexpressed in many cancer cases with collagen crosslinks that suggest a poor prognosis.^[^ ^20^ ^]^ LOX‐mediated collagen cross‐linking directly increases tumor cell proliferation, enhances metastasis, colonization, and growth, and manifests as increased metastasis in vivo.^[^ ^21^ ^]^ In breast cancer, the overexpression of LOX promotes invasion, metastasis, and EMT.^[^ ^22^ ^]^ Even in the absence of biochemical factors, alterations in collagen crosslinking status and ECM stiffness could induce the invasive behavior of the oncogene‐initiated epithelium.^[^ ^23^ ^]^ In addition, altered collagen crosslink types such as elevated proportions of hydroxylysine aldehyde‐derived collagen crosslinks, could promote tumor cell invasion.^[^ ^24^ ^]^

#### 2.2.3 Biomechanics Stimulate Stiffness

Another process easily ignored is that biomechanical cues could increase the stiffness of the ECM. For example, elevating matrix deformation could increase the resistance of crosslinked ECM, a process called strain stiffening.^[^ ^25^ ^]^ Contractile forces exerted by actomyosin in CAFs in the TME also lead to stiffening of the ECM.^[^ ^26^ ^]^ In addition, other nonmatrix factors, such as elevated cell density due to tumor growth and increased interstitial fluid pressure, also contribute to the increase in ECM stiffness.^[^ ^16c^ ^]^

#### 2.2.4 Cellular Consortiums and ECM Remodeling

The stiffening of the ECM is also the result of complex multidirectional interactions between cancer cells and various cells in the TME, such as CAFs, infiltrating immune cells, and tumor‐associated adipocytes^[^ ^27^ ^]^ (Figure 1). CAFs were deemed to be major contributors to ECM stiffness and degradation,^[^ ^28^ ^]^ which increased ECM stiffness mainly by increasing collagen deposition, overexpressing LOXs, and aligning the fibronectin in matrix.^[^ ^29^ ^]^ Hypoxia is another factor to promote stiff ECM formation in CAF‐associate tumor progression.^[^ ^30^ ^]^ Matrix metalloproteinases (MMPs) secreted and activated by tumor cells and CAFs also could promote basement membrane (BM) degradation and tumor cell migration.^[^ ^31^ ^]^ Additional, Tumor‐associated macrophages (TAMs) have been found to play a role in deposition, cross‐linking, and linearization of ECM collagen fibers. TAMs could up‐regulate the synthesis and assembly of collagen types I, VI, and XIV in other cells in tumor region.^[^ ^32^ ^]^ A subset of CD163/RELMα positive TAMs was also found to actuate stromal cell‐mediated collagen crosslinking, ECM stiffening, and tumor aggression.^[^ ^33^ ^]^ Besides CAFs and TAMs, the role of other TME infiltrated cells, such as cancer‐associated adipocytes, in promoting the expression of fibronectin was gradually exposed.^[^ ^34^ ^]^

### 2.3 The Mechanism of ECM Stiffness Effects on Cancer

ECM stiffness transfers mechanical signals from extracellular to intracellular through mechanotransduction pathways. In different types of cells, the mechanical transduction pathways that play a role in responding to ECM stiffness have both universality and individuality. Some signaling molecules, such as integrins, YAP, and ROCK have been found to work in different types of cells.^[^ ^6b^ ^]^

#### 2.3.1 Tensegrity Model

Tensegrity is a system that provides structural stability through imposing a tensile prestress in compressive and tensile manners.^[^ ^35^ ^]^ The cellular molecular regulation and cellular mechanotransduction could be explained via using tensegrity to structure cells themselves. For example, the cells were significantly sensitive to mechanical forces between the cytoskeleton and the ECM under the instruction of tensegrity model. Microfilaments in the cytoskeleton acted as tension units pulling cell membrane toward nucleus, and such tension was balanced by microtubules and integrins‐anchored ECM.^[^ ^36^ ^]^ Own to such internal tensile stress, slender mechanical signals in ECM could be captured and transmitted into cells.^[^ ^37^ ^]^ Along with the ECM changes, the cells transmitted mechanical signals into the inside of living cells to induce mechanotransduction and, and then the cells would occur different biological responses including cells proliferation and differentiation, gene expression, and spreading.

#### 2.3.2 Membrane Proteins act in Mechanical Transduction

In general, integrins are the starting point at which cells sense mechanical signals. integrins activate downstream molecules and regulate gene transcription through focal adhesion (FA), thus responding to the mechanical environment. Integrins are made up of two subunits, α and β, whose N‐terminal domains harbor the ECM ligand‐binding site, and the cytoplasmic tails of integrins act as the nucleation center for protein‐protein interactions between integrins and intracellular proteins.^[^ ^38^ ^]^ These subunits can transmit mechanical and chemical signals between the ECM and the cell interior by recruiting adaptor molecules and signal‐transducing molecules, including FA kinase (FAK) and Src family kinase (SFK), forming FA and further activating downstream pathways,^[^ ^39^ ^]^ such as YAP/TAZ, Rho/ROCK, and other signaling molecules mentioned in a later section. In addition to FA, integrins can also form adherens junctions (AJs), another kind of mechanical receptor sensing intercellular force.^[^ ^40^ ^]^ However, since most tumor cells are anchorage‐independent and gradually lose the expression of AJs, tumor cells rely mainly on the activation of the FA signaling pathway to transmit mechanical signals.^[^ ^41^ ^]^

Notably, although there is a perspective that discoidin domain receptors (DDRs) play a key role in perceiving changes in ECM stiffness,^[^ ^42^ ^]^ the DDR acts as an “effector” rather than a “receptor” in ECM stiffness mechanical transduction. In other words, the stiffness of the ECM leads to the alteration of DDR expression through the mechanical transduction signaling pathway, and then affects the cell behavior.^[^ ^43^ ^]^ There is still insufficient evidence to prove that DDR can directly sense the mechanical signals caused by matrix stiffening. Considering that the ligands of DDRs are collagens rich in stiff ECM regions,^[^ ^43b^ ^]^ further study could focus on the direct relationship between ECM stiffness and DDRs.

#### 2.3.3 The Response of Intracellular Molecules to ECM Stiffness

The transcription factors YAP and TAZ are downstream molecules in the Hippo pathway, which is a canonical pathway regulating multiple cellular functions in embryonic development. YAP/TAZ has also been revealed to have a crucial role in the mechanical transduction of cancer cells, by which cells respond to ECM stiffness.^[^ ^44^ ^]^ ECM stiffness signaling could inhibit LATS1/2 through integrins/FAK/Src, thus activating YAP/TAZ.^[^ ^45^ ^]^ In cancer cells, YAP/TAZ has been found to act in a Hippo‐independent manner requiring the integrity of the actin cytoskeleton, that is, directly activated by mechanic stimulation through integrins/Src or by forces acting on cell adhesions through AJs.^[^ ^45^, ^46^ ^]^ Activated YAP/TAZ moves from the cytoplasm to the nucleus and binds to enhancer elements via the transcription factor TEA domain family members (TEADs), thus activating downstream gene transcription.^[^ ^44^, ^47^ ^]^ For instance, YAP interacts with TEADs to promote transcription of thrombospondin (THBS1), which is the upstream gene of FAK, and thereby promotes phosphorylation of FAK and FA formation, leading to an invasive phenotype.^[^ ^48^ ^]^ Meanwhile, there is much evidence had been proved that YAP/TAZ is related to EMT in tumor cells such as breast cancer and nonsmall cell lung cancer.^[^ ^44^, ^49^ ^]^

The Rho and downstream Rho‐associated coiled‐coil protein kinase (ROCK) signaling pathways also play a crucial role in the regulation of cell‐matrix mechanical transduction. Extracellular mechanical signals aggregate integrins and activate Rho/ROCK via FAK and ERK.^[^ ^15^ ^]^ Activated Rho/ROCK increases the phosphorylation level of myosin light chain, resulting in an increase in myosin‐contractile force,^[^ ^50^ ^]^ and mechanical signals are transmitted to the nucleus through the polymerization of actin, triggering the nuclear translocation of myosin‐related transcription factor A (MRTF‐A).^[^ ^51^ ^]^ MRTF‐A, also known as MKL1 and MAL, is the coactivator of serum response factor (SRF). By binding the serum response element (SRE), the MRTF‐SRF complex activates gene transcription.^[^ ^52^ ^]^ The SRF/MRTF‐A pathway is associated with actin dynamics and the transcriptional regulation of a variety of EMT‐associated genes, such as Snail and Slug.^[^ ^53^ ^]^ Matrix stiffness could control the subcellular localization of MRTF‐A, which contributes to myogenic characterization during EMT.^[^ ^54^ ^]^

## 3 Mechanotransduction of TME on EMT

### 3.1 EMT in Cancer

EMT is a form of epithelial plasticity that plays an essential role in embryonic development, tissue regeneration, wound healing, fibrosis, and cancer.^[^ ^55^ ^]^ In EMT, epithelial cells lose E‐cadherin‐mediated cell‐cell adhesions, converse the polarity apical‐basal to front‐rear, change to elongated morphology, and display increased motility.^[^ ^56^ ^]^ In addition to these phenotypic shifts, cells exhibit alterations in gene expression, including upregulation of a variety of transcription factors such as Snail, Slug, and Twist, loss of epithelial markers such as E‐cadherin and zonula occludens protein‐1 (ZO‐1), and gain of mesenchymal markers such as N‐cadherin and vimentin.^[^ ^56^, ^57^ ^]^ EMT is induced mainly by the TGFβ pathway, the Wnt Signaling, the Notch Pathway, and signals from tyrosine kinase receptors, among which TGFβ signaling is a primary inducer of EMT.^[^ ^58^ ^]^ TGFβ‐induced activation of the receptor complex leads to the activation of Smad2 and Smad3 through direct C‐terminal phosphorylation by TβRI. Phosphorylated Smad2 and Smad3 then form trimers with Smad4 and translocate into the nucleus, where they associate and cooperate with DNA‐binding transcription factors to activate or repress target gene transcription. Consequently, Smad2 and Smad3 function in cooperation with Smad4 as TGFβ‐induced transcription regulators.^[^ ^59^ ^]^

In cancers, coexpression of epithelial and mesenchymal markers is associated with poor prognosis.^[^ ^10c^ ^]^ Although the role of EMT in tumors is currently controversial, a consensus has been reached that cancer cells in tumors experience “incomplete” or “partial” EMT. The mesenchymal‐like phenotype of cancer cells is associated with tumor growth, invasion, metastasis, and resistance to treatment.^[^ ^60^ ^]^ The loss of E‐cadherin in cancer cells impairs the balance between cell‐cell adhesions and integrins‐mediated cell‐ECM adhesion, resulting in the occurrence of invasion and initiating spreading to distant organs.^[^ ^61^ ^]^ Upon arrival, mesenchymal‐like cells revert to showing epithelial characteristics, thus restoring their ability to proliferate and undergo epithelial growth at remote organ sites. In addition, EMT‐activating transcription factors (EMT‐TFs) have been found to have a nonclassical effect in tumors, maintaining stem cell properties and increasing tumorigenicity, linking them to cancer stem cells.^[^ ^56^, ^62^ ^]^

### 3.2 Effects of ECM Stiffness on EMT

Despite the existence of universal receptors, the effects on EMT of different tumors vary due to the different downstream molecules of mechanical transduction pathways in different tumors. Here, we summarized recent reports focusing on the biological functions and underlying molecular mechanisms of ECM stiffness in regulating EMT of tumor cells in different types of cancer to explore new targets for cancer treatment.

#### 3.2.1 Breast Cancer

Breast cancer is the most common and fatal cancer among women in most regions of the world.^[^ ^63^ ^]^ According to the latest data released by IARC in 2020, the number of new cases of breast cancer reached 2.26 million, overtaking lung cancer (2.21 million) for the first time to become the world's most common cancer.^[^ ^64^ ^]^ Early‐stage studies on the effects of matrix stiffness in tumorigenesis were conducted in breast cancer, where increased tumor stiffness could lead to EMT, invasion, and metastasis of tumor cells.^[^ ^15^ ^]^ Mammary epithelial cells (MECs) form and remain spherical under normal conditions but begin to lose epithelial characteristics and acquire mesenchymal morphology in a stiff matrix, not exhibiting memory‐like behavior.^[^ ^65^ ^]^ The same phenomenon was also confirmed in vivo: migration from the injection site was increased with dynamic stiffening, thereby reducing the tumor load.^[^ ^66^ ^]^ In theory, stiffness‐mediated EMT was not a cellular autonomic process, but mechanosensing was indispensable.^[^ ^13^ ^]^

Independently, ECM stiffness could induce breast cancer cell EMT through mechanical transduction. The EMT‐inducing transcription factor TWIST1, which is retained in the cytoplasm through its interaction with Ras GTPase‐binding protein (G3BP2) on a soft matrix, was found to undergo nuclear transversions in a stiff matrix, by releasing TWIST1‐G3BP2 binding, thus driving EMT and metastasis.^[^ ^9a^ ^]^ Further study found that high ECM stiffness led to ligand‐independent phosphorylation of the ephrin receptor (EPHA2), which requires ERK and RSK to recruit and activate LYN. Then, activated LYN phosphorylated TWIST1 to release TWIST1 from G3BP2 into the nucleus, and sequentially initiates EMT. This process is independent of cell shape, cell polarity, and adhesion connections, where integrins are not directly involved in.^[^ ^67^ ^]^ In addition, a stiff matrix has been demonstrated to be able to stabilize the assembly of an actin scaffolding complex and control the subcellular localization of MRTF‐A, which is a regulator of cytoskeletal protein expression.^[^ ^68^ ^]^ The actin/MRTF‐A signaling pathway is specifically involved in the higher proliferation and myogenic characterization of the cells that undergo EMT, while MRTF‐A depletion is also shown to impact vimentin expression in EMT‐established cell lines.^[^ ^57^, ^69^ ^]^ In particular, epithelial cell transformation into fibroblasts and/or myofibroblasts could promote ECM component deposition, fiber formation, and further increase tissue stiffness, creating positive feedback.^[^ ^57b^ ^]^

Many stimulants, including biochemical and physical factors in the microenvironment could induce EMT together with matrix stiffness. For example, TGFβ, a classic ligand in EMT, has been found to interact with matrix stiffness. Matrix stiffness regulated this effect by regulating the PI3K/Akt pathway rather than Smad to switch the effect of TGFβ, which induced breast cell apoptosis in the soft matrix but EMT in the stiff matrix.^[^ ^9b^ ^]^ Breast cancer cells cultured on aligned fibers showed upregulated expression of metastasis‐related EMT genes, and elongated spindle‐like morphology rather than flat stellar shape in messy fibers. Cell alignment led to increased cell tension, which might lead to the upregulation of TGFβ1, which in turn promotes EMT.^[^ ^65^ ^]^ A stiff ECM is often associated with the upregulation of LOX‐2 expression.^[^ ^70^ ^]^ As a potential mediator, LOX combines mechanotransduction oncogenic signaling through TGFβ1. In normal MECs, LOX inactivation did not affect TGFβ1 to activate Smad2/3, while in malignant MECs, inhibition of LOX activity weakened the activation of P38 MAPK by TGFβ, suggesting that inactivating LOX function might prove effective in reducing TGFβ1‐stimulated breast cancer progression.^[^ ^71^ ^]^ In addition to the ECM remodeling function, the LOX‐like protein LOXL2, was found to be associated with increased expression of the EMT regulatory transcription factor Snail1 and other cytokines, which promotes the formation of a premetastatic niche.^[^ ^72^ ^]^ Activated epidermal growth factor receptor (EGFR) signaling pathway and matrix stiffening could also promote EMT and stemness and then upregulate the expression of PD‐L1, which is associated with cancer cells escaping immune elimination and multicellular aggregates.^[^ ^73^ ^]^ The stiffness of the ECM with MMP‐3 could control the membrane localization of Rac GTPases, making Rac1b and NADPH oxidase form a complex and then promoting the production of reactive oxygen species, snail expression, and EMT, whereas a soft matrix would suppress this process.^[^ ^74^ ^]^

Meanwhile, physical factors such as hypoxia, ECM topography, and defects of the BM also played a role in inducing EMT with ECM stiffness. Hypoxia and stiffer substrate (20 kPa) could synergistically induce phenotypic changes, apoptosis, and EMT in MCF‐7 cells.^[^ ^75^ ^]^ The topography and stiffness of the matrix have synergetic effects on the cell morphology and EMT, and the expression of vimentin is enhanced in highly oriented fiber alignment.^[^ ^76^ ^]^ Defects of BM, which commonly occur in cancer, can trigger EMT in normal epithelial cells. Stress fiber formation on the soft col‐IV matrix rises after defect‐induced EMT, whereas the process becomes more rapid and concurrent with EMT on a stiff matrix, which in turn enhances cellular mechanoactivation.^[^ ^77^ ^]^

Interestingly, soft matrix and low cytoskeletal tension may promote EMT in breast cancers, which corresponds to the clinical finding that very low mammographic density (<10%) indicates poor prognosis and histological tumor grade.^[^ ^78^ ^]^ Overexpression of oncogene H‐Ras could cause MECs to be insensitive to stiffness, causing partial diffusion on a physiologically healthy stiff substrate, showing cells in elongated mesenchymal morphology, interrupted BM, and the nuclear localization of TWIST1. This diffusion is driven by ERK activation rather than typical mechanosensitive pathways such as YAP and TGFβ or myosin contraction.^[^ ^79^ ^]^ In addition, an increase in matrix stiffness leads to an increase in cytoskeletal tension, which downregulates SOX4, a transcription factor maintaining the mesenchymal phenotype of breast cancer cells, and downstream mesenchymal marker expression, while the expression of other EMT‐related transcription factors (EMT‐TFs) remains unaffected. TRPM7, a regulator involved in mechanical sensory processes, could reduce cytoskeletal tension to promote the expression of SOX4.^[^ ^80^ ^]^ The same phenomenon can be seen in other types of cancer cells,^[^ ^81^ ^]^ suggesting that matrix stiffness is involved in tumor EMT through a very complex mechanism of action.

#### 3.2.2 Lung Cancer

Lung cancer is the most prevalent cancer in men and the leading cause of cancer death, representing close to 1 in 5 cancer deaths in the world.^[^ ^63^, ^64^ ^]^ Clinical research has found that the incidence and mortality of lung cancer are higher in patients with idiopathic pulmonary fibrosis than in patients with emphysema.^[^ ^82^ ^]^ In principle, a stiff matrix could induce spontaneous EMT in alveolar epithelial cells. Increased matrix stiffness could independently promote cell proliferation and EMT by activating integrins/FAK‐mediated mechanotransduction, which selectively upregulates the EGFR and hepatocyte growth factor receptor (c‐Met), which is more sensitive than EGFR in response to stiffness‐mediated EMT, and Snail expression. This response could be eliminated by supporting fibrinogen fragments FnIII9p10, which engage integrins a3 and a5.^[^ ^83^ ^]^

In addition, substrates‐induced EMT is associated with integrin‐mediated TGFβ activation and is dependent on Rho/ROCK signaling.^[^ ^84^ ^]^ Increased cell contraction is indispensable, and suppression of cell contractility is sufficient to suppress the response.^[^ ^85^ ^]^ Although the lung epithelium shows an increase in EMT when cultured in stiff ECM, tumor cell EMT was not determined by ECM stiffness in the A549 cell line, while associated mainly with TGFβ.^[^ ^86^ ^]^ Decreased levels of phosphorylated FAK and paxillin were found in lung cancer cells on stiffer substrates, which means there are other mechanotransduction pathways worded differently from alveolar epithelial cells. DDR 2 was found to be involved in TGFβ‐induced EMT progression in lung adenocarcinoma.^[^ ^43a^ ^]^ A recent study showed that a stiff matrix upregulated c‐Myb acetylation by p300, which appeared to be necessary for c‐Myb and LEF1‐mediated DDR2 expression. The c‐Myb‐DDR2 axis is essential for the proliferation of cancer cells and the expression of EMT marker genes on a stiff substrate, which is a necessary condition for the regulation of lung cancer cell EMT and invulnerability.^[^ ^87^ ^]^

In addition, EMT could also be activated by other physical factors with a stiff matrix. Matrix topography and stiffness could induce EMT directly independent of exogenous cytokines, and the topography is necessary for stiffness‐mediated EMT activation, which depends on the activation of the PI3K/Akt signaling pathway.^[^ ^88^ ^]^ Changes in matrix stiffness and cell adhesion ligand concentration also regulate the loss of epithelial morphological characteristics caused by exposure to TGFβ such as changes in EMT marker gene expression, and reduction of Mir‐200 level consistent with EMT, regardless of matrix composition.^[^ ^89^ ^]^ Synergies between other components of the TME such as TAMs and matrix stiffness should also be considered. The coculture of tumor cells with M0 macrophages could induce an alternative activated M2‐like phenotype. The presence of tumor‐associated M2c macrophages and the stiffness of the ECM together contribute to the invasive phenotype and regulate the expression of specific EMT‐related genes. Under high stiffness, the M2c upregulated CDH2, MYC, and TWIST1, which are proliferation‐ and invasion‐ associated genes, whereas no changes in the expression of genes related to EMT are observed under low stiffness conditions.^[^ ^90^ ^]^

#### 3.2.3 Pancreatic Cancer

Pancreatic cancer, with a 5‐year relative survival rate, is less than 4%, is one of the most fatal cancers and responds very poorly to chemotherapeutic agents. Pancreatic cancer is also one of the hardest human solid cancers, with highly fibrotic and crosslinked ECM.^[^ ^91^ ^]^ Tissue stiffening in pancreatic cancer tissues promoted the characteristics of EMT, including elevated vimentin expression, and decreases in E‐cadherin expression.^[^ ^92^ ^]^ Growth factor signaling is combined with mechanical signaling in pancreatic cancer EMT. Bone morphogenetic proteins (BMPs) are members of the TGFβ family. Mechanically sensitive YAP1 played a key role in BMP4‐induced EMT and invasion, where BMP4 promoted nuclear accumulation of YAP1, and a stiff matrix is indispensable.^[^ ^93^ ^]^ Specific activation of GPER inhibits YAP activation in cancer cells through the GTPase Ras homologous family member A (RhoA), thereby inhibiting mechanical transduction, cell contraction, and EMT.^[^ ^94^ ^]^ Nuclear localization of β‐catenin has also been found in a stiff matrix, but it occurred in a binary manner, which means that other pathways reacted in stiffness‐mediated EMT.^[^ ^92^ ^]^ In addition to growth factor signaling, ECM proteins also participate in stiffness‐mediated EMT. Pancreatic ductal adenocarcinoma (PDAC) exposure to fibrillar interstitial matrix could drive EMT, while exposure to the nonfibrillar BM promotes epithelial behavior.^[^ ^95^ ^]^ Although fibrillar type I collagen is found to increase the expression of specific mesenchymal markers, PDAC morphology and phenotypic heterogeneity mainly rest on matrix fibril density and associated stiffness.^[^ ^95^ ^]^ Hyaluronic acid (HA), an essential tumor matrix, and matrix stiffening synergistically promoted invasive and stiffness‐mediated EMT phenotypes in PDAC cells.^[^ ^96^ ^]^

#### 3.2.4 Liver Cancer

Liver cancer or hepatocellular carcinoma (HCC), the second leading cause of death among men, is highly correlated with cirrhosis, with up to 90% of HCC occurring in terminal fibrosis or cirrhosis.^[^ ^97^ ^]^ Elevated liver stiffness has become a nonnegligible clinicopathological parameter for the pathological grading and prediction of HCCs prognosis.^[^ ^98^ ^]^ The soft environment promotes the differentiation phenotype of hepatocytes, and the increased matrix stiffness is related to the dedifferentiation of cells to the mesenchymal phenotype.^[^ ^99^ ^]^

In the presence of TGFβ1, hepatocytes undergo EMT or apoptosis, depending on different matrix stiffness: culture on stiff collagen results in EMT, while hepatocytes cultured on soft collagen matrix experienced programmed cell death. This response is Snail independent.^[^ ^100^ ^]^ A stiff matrix with immobilized‐TGF (i‐TGF) is the only condition to induce the mesenchymal phenotype, and the absence of either would result in the abolition of the process. In a stiff matrix, i‐TGF stimulates TGF‐β1 receptor (TβRI) expression, and i‐TGF‐TβRI interactions promote stiffness sensing, resulting in elevated expression of β1 integrins and stimulation of the β1 integrins/vinculin/p‐FAK pathway, which notably activates PI3K downstream, resulting in a mesenchymal phenotype and accelerated migration.^[^ ^101^ ^]^

However, another study found that higher matrix stiffness as an initiator independently triggers EMT in HCC cells. The study found three signaling pathways converging on Snail expression participating in stiffness‐mediated EMT, one of which was the increased autocrine activity of TGFβ1, which may correspond to previous research. A stiff matrix stimulates the membrane localization of S100A11 via integrins, leading to the interaction between S100A11 and NADPH oxidase, which promotes the production of ROS. ROS could increase the hypermethylation of the E‐cadherin promoter and have a positive relationship with EMT. Under the stimulation of high stiffness, the miRNA‐24‐3p in HCC cells is lost, but the target gene Furin is increased, which depends on integrins β1. Elevated Furin enhances the autocrine activity of TGFβ1, further increasing Smad2/3 phosphorylation and snail expression, and triggering the EMT. Moreover, stiff stimulation could upregulate the phosphorylation levels of Raf1 and elF4E through integrins α5 or β1, further regulating the expression of Snail and MMP‐3.^[^ ^9c^ ^]^

In addition to TGFβ signaling, increasing matrix stiffness markedly upregulates C‐X‐C chemokine receptor type 4 (CXCR4) expression in HCC cells, and CXCR4 decreases the levels of ubiquitin domain‐containing protein 1 (UBTD1), which functions in the proteasome‐dependent degradation of YAP. Downregulation of UBTD1 decreases YAP ubiquitylation and results in the activation of downstream signaling and YAP‐targeted genes, resulting in cell proliferation, EMT, and stemness on the stiff matrix, correlated with malignant prognostic features and overall survival.^[^ ^102^ ^]^

#### 3.2.5 Ovarian Cancer

Despite the effective treatments for localized ovarian cancer, this cancer remains the most fetal gynecological malignant cancer, due to its hysteretic diagnosis after metastasis.^[^ ^103^ ^]^ BMP4 could induce OvCa429 cell EMT in stiff substrates, a process that depends on the matrix stiffness and mechanical reactivity of YAP1. The kinase activity of CDK8, which is associated with EMT genes and promotes EMT‐related invasion, also plays an important role in this process. Targeting CDK8/19 inhibits EMT‐related transcriptional changes and invasion, which depend on the integration of growth factor signaling (BMP/SMAD1) and machinery (YAP1) in the tumor. A direct association between CDK8 and EMT‐related transcription factors is found in patients.^[^ ^93^ ^]^ Whereas, metastatic ovarian cancer cells (OCCs) exhibit mechanical sensitivity and preferentially adhere to the soft microenvironment. Enhanced malignant phenotypes and EMT occurred in cancer cells after culture on a soft substrate, and this mechanical tropism is controlled through the Rho/ROCK pathway. In less‐invasive OCCs, mechanosensitivity is decreased, which means that mechanical cues are the key determinant of ovarian cancer spread.^[^ ^104^ ^]^

#### 3.2.6 Other Cancers

Osteosarcoma is the most common solid tumor in children. One of the most significant differences between bone ECM and nonmineralized tissue is that bone ECM is approximately 10^5^ to 10^6^ times harder.^[^ ^105^ ^]^ Matrix stiffness could promote EMT and further promote tumor cell invasion by regulating the nuclear translocation of cytoskeleton and MRTF‐A, as the epithelial marker E‐cadherin on the stiff matrix decreases and the mesenchymal markers Vimentin and Fibronectin increase.^[^ ^106^ ^]^

Gastric cancer is the third leading cause of cancer death. Gastric cancer cells lose the expression of the standard CD44 subtype (CD44s) and obtain expression of VARIANT 6 of CD44 (CD44v6) in stiff 3D culture, accompanied by upregulation of EMT, metabolism, and angiogenesis‐related genes.^[^ ^107^ ^]^

Colorectal cancer ranks second in terms of mortality. Colorectal cancer cells cultured under appropriate matrix stiffness conditions can produce E‐R transformation (epithelial‐like phenotype cells to rounded, separated cells), and the E‐cadherin decreases significantly. R cells are more aggressive and more than 90% of R cells express tumor stem cell markers, suggesting that appropriate matrix stiffness can promote the transformation of colon cancer tumors into tumor stem cells. Notably, this change in colorectal cancer cells is irreversible.^[^ ^108^ ^]^

**Figure 2:**
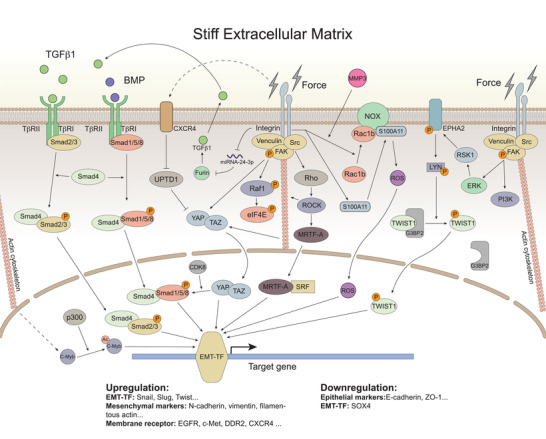
The mechanical transduction pathway network plays a role in stiffness‐mediated EMT by regulating target genes. The TGFβ family activates downstream pathways through the TβR to promote EMT. Through mechanical transduction pathways, ECM stiffness activates different downstream molecules and then regulates the activation of EMT‐TF, which controls the downstream gene transcription such as upregulating mesenchymal markers, elevating cell membrane receptor expression, and downregulating epithelial markers. The dotted line indicates that there is a relationship in upregulation, but the direct action needs further confirmation.

**Figure 3:**
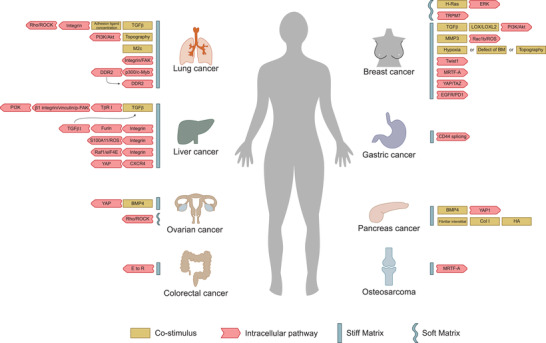
Inducers and intracellular pathways involved in stiffness‐mediated EMT in different types of cancers. The mechanotransduction pathways in different types of cancers are not the same. In most cases, a stiff ECM can promote EMT in cancer cells, while in H‐Ras‐transformed MCF10A cells and metastatic OCCs, it is a soft matrix that promotes EMT.

In oral squamous cell carcinoma (OSCC) patients, increased collagen tissue predicts poor prognosis. On stiff substrates, highly invasive OSCC cell lines contained a low E‐cad to N‐cad ratio (InvH/E:NL) and low invasive cells have the opposite E/N (InvL/E:NH). Cells with different degrees of invasion respond differently to matrix stiffness. Retaining higher plasticity in a stiff matrix, InvL/E:NH cells gradually acquire mesenchymal characteristics and begin to migrate rapidly after prolonged exposure, suggesting that cells can be mechanically regulated (**Figures** **2** and **3**).^[^ ^109^ ^]^

## 4 Applicable Culture Systems Mimicking the Natural ECM in Cancer Research

With the influence of physical factors on the cells gradually becoming apparent, an applicable culture system is particularly significant for in vitro experiments. The 2D cell culture is the most widely used method in the laboratory because it is easy to operate, inexpensive, and suitable for long‐term culture. However, unlike normal cells surrounded by other cells and ECM in vivo, cells grow in the form of a single layer on a flat plastic surface under 2D culture, which is much harder than the physiological environment, so that cells are forced to be flat and elongate. Without the spatial dimension, 2D‐cultured cells lack the proper physical factors, resulting in a different response to external stimuli than under physiological environments. In addition, the gene expression of tumor cells in 2D culture is different from that of tumor cells in vivo. Therefore, cells grown under nonphysiological monolayer conditions cannot present drug resistance derived from the 3D structure and the complex TME, resulting in a high failure rate for drug development.^[^ ^110^ ^]^

In recent years, the appearance of 3D culture systems has overcome the deficiencies of 2D culture, providing the possibility to mimic the natural environment, especially to explore the role of biomechanical signals. Following this, the proximate ECM stiffness cultivation systems were reviewed.

### 4.1 Traditional ECM Culture System

The mainstream 3D culture system can be divided into two types: scaffold‐based culture and nonscaffold‐based systems.^[^ ^110c^ ^]^ Cancer cells in scaffold‐based culture systems are supported mainly by hydrogels derived from natural or artificial materials. Nonscaffold‐based systems are also called multicellular tumor spheroid models (MCTSs), whose matrices are produced by cancer cells rather than exogenous support.^[^ ^110c^ ^]^ Conventional MCTS physical properties are poorly regulated due to insufficient ECM deposition,^[^ ^111^ ^]^ but methods have emerged in recent years to remedy this defect of MCTSs. For example, cancer cells can be integrated with cellular and noncellular interstitial components such as fibroblasts, endothelial cells, and ECM, forming a heterotypic 3D spheroid model to increase the mechanical properties of the ECM.^[^ ^111^ ^]^ Another approach is combing with scaffolds systems. Cancer spheroids can be combined with a collagen and alginate mixed hydrogel system to study the effect of matrix stiffness on tumor cells.^[^ ^112^ ^]^ or with a polyethylene glycol (PEG)‐heparin‐based 3D microenvironment.^[^ ^113^ ^]^

For the scaffold, different materials have their own characteristics. Tissue‐derived materials are closer to the physiological environment and have better biocompatibility and cell adhesion, but the physical properties of tissue‐derived materials are less adjustable, and there may be differences between batches of different products. Decellularized ECM (dECM) is fabricated by removing cellular components from the tissue while leaving most ECM structures. In comparison to the onefold ECM protein scaffold currently used in cell culture systems, dECM retains the unabridged biochemical complexity of natural tissues, providing the closest to physiological conditions and a tissue‐specific microenvironment.^[^ ^114^ ^]^ However, the lack of methods to control the mechanical properties of dECM materials limits their application in exploring organizational structure and mechanical properties.^[^ ^115^ ^]^

Natural ECM protein materials, such as collagen I, Matrigel, and fibrin, were initially used to compose protein fiber structures, which could best simulate the internal environments. The widely used material is Matrigel which can be polymerized to form a biologically active 3D matrix, simulating the structure, composition, physical properties, and function of the in vivo BM.^[^ ^116^ ^]^ As Matrigel is derived from EHS tumors, it may contain proteins produced by tumorigenic cell lines.^[^ ^117^ ^]^ Collagen and fibrin are commonly used ECM components that have good characteristics of cell adhesion, proliferation, and distribution. The fibrinogen structure of collagen and the ability to form reasonable mechanical property networks in vitro make it the gold standard for the reconstruction of 3D cell culture scaffolds.^[^ ^118^ ^]^ Fibrin scaffolds are similar to natural EMT in composition and structure, have low antigenicity and immunogenicity and can exert a certain stimulation on cells.^[^ ^119^ ^]^ However. the weakest point of collagen and fibrin is the low mechanical strength (elastic moduli range from 100 to 150 Pa) and easy degeneration.^[^ ^119^, ^120^ ^]^ Moreover, because the stiffness alteration of those materials usually occurs with other material properties, including surface chemistry, morphology, and adhesive ligand availability changing synchronously, it is difficult to study the effect of ECM stiffness independently.^[^ ^121^ ^]^ Thus, a culture system that could change the physical features separately should be established.

Hyaluronic acid (HA), a glycosaminoglycan that is abundant in the ECM, has traditionally been considered a biological “goo” widely used in tissue engineering and cell culture. HA may be an important matrix ingredient in the study of cancer and angiogenic response due to its rich content and upregulation in many tumor tissues. However, the mechanical properties and adhesion of HA are poor.^[^ ^122^ ^]^

Alginate is a natural anionic polymer extracted from brown algae and has been widely used in many biomedical applications because it is relatively low cost, low toxicity, biocompatible, and can mildly gelate with the addition of divalent cations (such as Ca^2+^). Likewise, alginate is bioinert because it lacks natural ligands interacting with mammalian cells.^[^ ^123^ ^]^

Synthetic materials have stable properties and easily altered stiffness but lack adhesion ligands. Polyacrylamide (PA) hydrogels are a widely used synthetic polymer in molecular biology and can also be used in cell culture. PA hydrogels are suitable for the study of mechanobiology, where the stiffness requires exact control. However, a major drawback of PA hydrogel is the high biotoxicity of hydrogel precursors so that PA hydrogel cannot encapsulate cells for 3D culture.^[^ ^12^ ^]^ Unlike PA, the unpolymerized components of PEG hydrogels are more biocompatible. PEG hydrogels are usually formed by mild photoinitiated polymerization techniques with the advantages including high viability of encapsulation, light polymerization ability, adjustable mechanical properties, and easily controlled scaffold structure and chemical compositions.^[^ ^124^ ^]^ However, PA and PEG are both generally considered biologically inert and are not conducive to cellular adhesion, making it difficult to search for the effects of bioactive molecules and adhesion ligands.^[^ ^12^ ^]^

### 4.2 Novel ECM Stiffness Culture Systems

**Figure 4:**
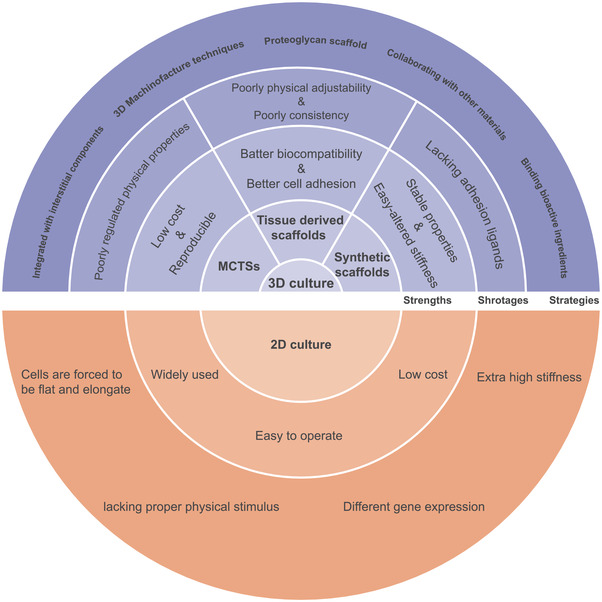
Diagram of 2D and 3D culture system. Different strategies to construct 3D culture systems for studying physical cues (upper part). The concentric circles represent the strengths, shortages, and strategies of different cultural systems from the inside out. More details are available in Table 2.

To ameliorate the defects of these materials, researchers produced new suitable cell culture systems by looking for natural biomaterials, providing on‐need bioactive ingredients, and constructing intelligent culture systems (**Figure** **4**).

#### 4.2.1 PG Based Scaffold

PG can carry out chemical modification and crosslink moieties or bioactive molecules more flexibly due to the plentiful functional groups on the polymer backbone, thus increasing cell adhesion and facilitating finding the effect of ECM stiffness and adhesion ligand density.^[^ ^121a^ ^]^ Gellan gum (GG), analogous to ECM mucopolysaccharide, is a bacterial exopolysaccharide. Silk fibroin originating from Lepidoptera is fibrous, similar to collagen I. Combining GG with silk protein can fabricate a hydrogel network allowing cell adhesion, and the stiffness can be controlled by modifying the mixing ratio of GG‐Silk.^[^ ^125^ ^]^ Chondroitin sulfate (CS) is a sulfated glycosaminoglycan (GAG) found in the ECM, and Chitosan is a commonly used biological material whose chemical structure is similar to that of GAGs, with the advantages of hydrophilicity, biodegradability, biocompatibility, and low immunogenicity. By adding CS to a chitosan scaffold, the biomaterial scaffolds appeared to have an effect similar to the effect of PG versican.^[^ ^126^ ^]^ Chitosan is cationic and can interact with anionic polymers to form polyelectrolyte complexes (PECs). PECs can provide the advantages of each polymer in the complex while hiding their respective weaknesses; thus, the culture system combining Chitosan with alginate is widely used.^[^ ^127^ ^]^

#### 4.2.2 Binding Bioactive Ingredients

Binding bioactive ingredients to hydrogels, such as integrins‐binding ligand RGD, is another way to improve cell adhesion ability. PEG hydrogels can be modified to render hydrogels bioactive and alter stiffness independently. Monoacrylate‐PEG‐succinimidyl carboxymethyl (PEG‐SCM) combines with MMP‐sensitive peptide GGGPQGIWGQGK (PQ) to form the cell‐degradable hydrogel backbone (PEG‐PQ‐PEG). PEG incorporates fibronectin‐derived Arg‐Gly‐Asp‐Ser peptide (RGDS), forming a scaffold pendant group (PEG‐RGDS), which promotes cell adhesion.^[^ ^89^ ^]^ Gelatin methacrylate (GelMA) has an RGD motif in its sequence, which permits cells to attach. Combining bioinert PEG diacrylate (PEGDA) gels with GelMA increases cell attachment, showing advantages in modulating anchorage‐dependent cell functions over nonadhesive materials. By changing the PEGDA and GelMA proportions, the stiffness and matrix ligand density can be manipulated independently without other property alterations.^[^ ^41^ ^]^ PEGDA can also be conjugated with fibrinogen. The thiol group at the cysteine site on the fibrinogen molecule reacts with the PEGDA polymeric chain in a Michael‐type addition reaction, forming PEG‐fibrinogen (PF). The Young's modulus of PF hydrogels can be altered by increasing the amount of PEGDA.^[^ ^128^ ^]^ PEG‐heparin based 3D model, whose mechanical properties could be altered independently without affecting ligand density, was fabricated by coupling Cysteine residues within the four‐arm PEG and maleimide‐modified heparin.^[^ ^113^ ^]^ In another study, Linear PEG‐disthiol (PEG‐SH) and MMP‐cleaved sequence (CGPQGIWGQC) were crosslinked to form a hydrogel network, where the cell adhesion peptides (CRGDS) were combined to promote cell adhesion.^[^ ^129^ ^]^

Alginate–RGD hydrogels could be fabricated though coupling the oligopeptide GGGGRGDSP to the alginate, which allowed cell adhesion.^[^ ^130^ ^]^ For one modified HA hydrogel system, The acrylate HA (AHA) was crosslinked with an enzymatically degradable peptide containing MMP1 and MMP2 sensitive sequences and two cysteines, and incorporated adhesion through RGD, in which thiol group was provided by cysteine to react with acrylate, forming a modular culture system.^[^ ^131^ ^]^ For another, HA polymers were firstly modified with aldehyde groups, which are cross‐linked with bis(oxyamine)‐ PEG via oxime ligation to control hydrogel mechanical properties, and then with methyl furan motifs, which are conjugated with maleimide‐functionalized bioactive peptides via Diels–Alder reaction to control hydrogel biochemical properties. By using such technique, a hydrogel culture system whose stiffness and bioactivity could be independently modulated was established.^[^ ^132^ ^]^

#### 4.2.3 Collaborating With Other Materials

Liver dECM was combined with GelMA to produce a photocrosslinkable solution, which was printed into hexagonal lobules close to the size of liver lobules using a rapid 3D bio‐printing technology based on DLP. Changes in stiffness can be easily controlled by changing the exposure time rather than altering the dosage of materials, thus eliminating the side‐effects of different concentrations or chemical components on cells.^[^ ^115^ ^]^

The fibrin scaffold system has also been exploited. Salmon fibrin can be used to fabricate flexible scaffold with non‐linear elasticity that is nontoxic, have low immunogenicity, and have low viral susceptibility.^[^ ^81^, ^133^ ^]^ Type I collagen Oligomer (IM) can rapidly polymerize to form highly interconnected, D‐banded collagen‐fibril networks, similar to tissues in vivo, and matrix stiffness could be systematically controlled by varying fibril densities.^[^ ^95^ ^]^

Another disadvantage of classical hydrogels is that the porosity varies with the stiffness of the material, which weakens the diffusion hydrogels of small molecules in a stiff matrix. A novel system based on an interpenetrating network (IPN) of alginate and Matrigel was set to overcome this problem. By enhancing calcium crosslinking rather than altering the polymer concentration, the IPN stiffness increased without changing the pore structure and ligand accessibility of IPN, and thus, it does not disturb diffusion.^[^ ^90^, ^120^ ^]^

#### 4.2.4 The “On‐demand” Stiffen Systems

During tumorigenesis, the ECM is undergoing an incessant transformation. Recent advances in materials have created dynamic or “on‐demand” systems, where cross‐linking is temporarily modulated via a stepwise crosslinking to achieve continuous or progressive cross‐linking or degradation, closer to in vivo tissue conditions. Common techniques include thermal‐activated calcium crosslinking, light‐mediated crosslinking, and enzymatic crosslinking. An alginate‐based hydrogel was dynamically stiffened by a temperature‐induced release of calcium.^[^ ^134^ ^]^ However, the potential adverse impact of nonphysiological temperature needs to be noted. Via radical polymerization, UV‐activated crosslinking can be precisely controlled over space and time. The methacrylated glycosaminoglycan hyaluronic acid (MeHA) hydrogel network was prepared using dithiothreitol as a cross‐linking agent, which was further cross‐linked by UV light after cell inoculation to the hydrogel. The final hydrogel stiffness could be dynamically modulated by UV exposure time or multiple UV exposures.^[^ ^13^, ^135^ ^]^ However, cells might be impaired by prolonged exposure to UV light but could be avoided by enzyme‐induced crosslinking. In an enzyme‐induced “on‐demand” system, gelatin was dually modified by norbornene and 4‐hydroxyphenylacetic acid. After thiol‐norbornene photopolymerization, the dityrosine crosslink was then catalyzed by tyrosinase. The crosslinking density and stiffness of the hydrogel were increased as needed, and the on‐demand stiffening reaction triggered by tyrosinase was realized (**Table** **2**).^[^ ^96^ ^]^

**Table 2:** Novel scaffold‐based culture systems used to study the role of ECM stiffness in cancers

	Culture system	Materials	Specialty	Cell type	Ref.
PG scaffold	GG−Silk Spongy‐like Hydrogel	GG and Silk protein	Combining GG with silk protein can fabricate the hydrogel network allowing cell adhesion, and the stiffness can be controlled by modifying the mixing ratio of GG‐Silk.	Osteosarcoma	^[^ ^125^ ^]^
	3D porous chitosan‐CS (C‐CS) scaffolds	Chitosan and CS	Through adding CS to a chitosan scaffold, the biomaterial scaffolds appear to have an effect similar to PG versican, and C‐CS scaffolds are a suitable culture platform in vitro for PCa.	Prostate cancer	^[^ ^126^ ^]^
	3D porous chitosan‐alginate (CA) scaffolds	Chitosan and Alginate	Chitosan is cationic and can interact with anionic polymers to form polyelectrolyte complexes (PECs). PECs can provide the advantages of each polymer in the complex, meanwhile hiding their respective weakness.	Prostate cancer glioma	^[^ ^127^ ^]^
Binding bioactive ingredients	Bioactive peptides modified PEG scaffold	PEG‐PQ PEG‐RGDS	The PEG hydrogels can be modified to render hydrogels bioactive and alter stiffness independently.	Lung cancer	^[^ ^89^ ^]^
	3D PEGDA/GelMA hydrogel matrix	PEGDA and GelMA	Gels incorporating GelMA have an RGD motif in the sequence and the ability to bind cells. Altering the ratio of PEGDA and GelMA permits manipulation of the matrix ligand density and stiffness, respectively, without changing other properties.	Osteosarcoma	^[^ ^41^ ^]^
	PEG‐fibrinogen (PF) hydrogel	PEGDA and Fibrinogen	The Young's modulus of PF hydrogels can be altered by increasing the amount of PEGDA.	Breast cancer	^[^ ^128^ ^]^
	PEG‐heparin‐based 3D model	PEG and Heparin	By coupling cysteine residues within the four‐arm PEG and maleimide‐modified heparin, the mechanical properties can be altered independently without affecting ligand density.	Breast cancer	^[^ ^113^ ^]^
	PEG‐SH scaffold hydrogel	PEG‐disthiol	Linear PEG‐disthiol (PEG‐SH) and MMP‐cleaved sequence (CGPQGIWGQC) are crosslinked and the cell adhesion peptides (CRGDS) can promote cell adhesion.	Brain tumor	^[^ ^129^ ^]^
	Alginate–RGD hydrogels	Alginate	Through coupling the oligopeptide GGGGRGDSP to the alginate to allow cell adhesion.	Osteosarcoma	^[^ ^130^ ^]^
	Modified HA hydrogel	HA	The acrylate HA is crosslinked with an enzymatically degradable peptide and two cysteines, and incorporates adhesion through RGD, forming a modular culture system.	Fibrosarcoma	^[^ ^131^ ^]^
		HA	By using two biorthogonal chemical strategies (oxime ligation and Diels–Alder reaction) within the same HA polymer backbone, the stiffness and bioactivity of the hydrogel can be independently modulated.	Breast cancer	^[^ ^132^ ^]^
Collaborating with other materials	3D bioprinted dECM scaffolds	dECM and GelMA	Liver dECM is combined with GelMA to produce a photocrosslinkable solution, which is printed into hexagonal lobules close to the size of liver lobules using a rapid 3D bioprinting technology based on DLP. Changes in stiffness can easily be controlled by changing the exposure time.	Liver cancer	^[^ ^115^ ^]^
	3D salmon fibrin gel	Thrombin‐activated purified fibrinogen	This mechanistic approach is useful for screening stem‐cell‐like cancer cells independently of stem cell markers.	Melanoma Ovarian cancer Liver cancer Lymphoma	^[^ ^81^, ^133^ ^]^
	3D matrices with type I collagen Oligomer (IM)	Col I oligomer and Matrigel	Oligomer can polymerize rapidly to form highly interconnected D‐banded collagen‐fibril networks, which are similar to the networks found in tissues in vivo.	Pancreatic cancer	^[^ ^95^ ^]^
	IPN 3D coculture hydrogel system	Alginate and Matrigel	Allowing alteration of ECM stiffness independently of composition and 3D architecture, the average pore size is similar for all the IPNs, therefore, not affecting diffusion.	Lung cancer Breast cancer	^[^ ^90^, ^120^ ^]^
	Collagen‐IV‐coated PA gel	PA and Collagen‐IV	Functionalized a layer of col‐IV to mimic BM‐like properties.	Breast cancer	^[^ ^77^ ^]^
Dynamic stiffness	Thermal induced crosslinking	Alginate	Temperature‐sensitive liposomes using encapsulated gold nanorods will release calcium or chelator when exposed to NIR light, resulting in alginate gelation and crosslinking.	/	^[^ ^134^ ^]^
	Photopolymerization	MeHA	Using dithiothreitol as a cross‐linking agent, the MeHA hydrogel network is further cross‐linked by UV light after cell inoculation to the hydrogel.	Breast cancer	^[^ ^13^, ^135^ ^]^
	Enzymatic crosslinking	Thiol‐norbornene and 4‐hydroxyphenylacetic acid‐modified gelatin	In norbornene and 4‐hydroxyphenylacetic acid‐modified gelatin, di‐tyrosine crosslink is then catalyzed by tyrosinase, realizing the on‐demand stiffening.	Pancreatic cancer	^[^ ^96^ ^]^

[Abbreviation] CS: Chondroitin sulfate; dECM: decellularized extracellular matrix; GelMA: Methacrylated gelatin; GG: Gellan gum; MeHA: Methacrylated glycosaminoglycan hyaluronic acid; PA: polyacrylamide; PEG: polyethylene glycol; PEGDA: Poly (ethylene glycol) diacrylate.

#### 4.2.5 3D Machinofacture Techniques

Electrospinning is a pre‐3D manufacturing technology to fabricate ECM stiffness system in the sub‐micron range with controllable mechanical properties and adjustable surface topography and chemical properties.^[^ ^65^, ^76^, ^136^ ^]^ Besides, micropillar substrates prepared by silicon etching and photolithographic technology could also be used to mimic the ECM structure,^[^ ^88^, ^137^ ^]^ these pre‐3D systems only mimic ECM in limited dimensional, the real biological function of cells in vivo is not realized.

The 3D bioprinting is a novel technology in the field of regenerative medicine that can achieve precise control of cells, biomaterials, and biomolecules.^[^ ^138^ ^]^ To date, some features and functions of cell‐printed structures are almost like the natural tissue. In oncology research, previous study had successfully created cervical cancer models by 3D‐printing HeLa cells,^[^ ^139^ ^]^ and another study had established a 3D‐bioprinted glioma stem cell model using modified porous gelatin/alginate/fibrinogen hydrogel to mock the ECM, achieving higher tumor cell survival and proliferation rates.^[^ ^140^ ^]^ Perfusion pipeline.^[^ ^141^ ^]^ The heterogeneous TME of glioblastoma patients could be reconstructed by 3D printing to build a drug screening platform.^[^ ^142^ ^]^ In combination with vascularization, metastasis model of breast cancer based on 3D cell printing was also successfully constructed.^[^ ^143^ ^]^

Besides, the selection of biological ink materials can provide controllable physical, chemical, and biological clues.^[^ ^138^ ^]^ By altering the printing parameters and the concentration ratio of GelMA to HA, different regions with different stiffness (glioblastoma multiforme region, acellular ECM region, and endothelial cell region) were printed to simulate the brain parenchyma under pathological or physiological conditions. With this model, biophysical cues were found to be associated with cancer cell behaviors, angiogenic potentials, and different molecular subtypes of glioblastoma multiforme.^[^ ^144^ ^]^ Another study altered the mechanical properties of the substrate by adjusting the ratio of collagen‐containing medium, sodium alginate, and gelatin. The intercellular communication was successfully reproduced by assembling the functional tunneling nanotube‐like cell projections.^[^ ^145^ ^]^

Especially, with tumor cell droplet as primary tumor, the natural hydrogel containing fibroblast as ECM, endothelialized microchannels as vascular conduits, and programmable release capsules as a source of chemical signals gradient, 3D printing made it possible to spatial‐temporal specifically control the signal molecule gradient in tumor models, so as to dynamically regulate cell behavior and simulate key steps in the progression of cancer.^[^ ^146^ ^]^

## 5 Conclusions and Outlook

**Figure 5:**
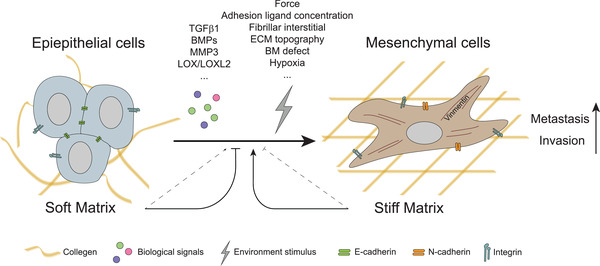
Stiffness‐mediated EMT occurs in cancer cells. ECM stiffness can independently act as a force cue or synergistically stimulate EMT in cancer cells with costimulators, including biochemical (such as TGFβ family, LOX family, MMP3) and physical factors (ECM topography, adhesion ligands concentration, hypoxia, etc.). In soft matrix cases, the loss of E‐cadherin in cancer cells impairs the balance between cell–cell adhesions and integrins‐mediated cell‐ECM adhesion, resulting in the occurrence of metastasis and invasion. The thin dotted line means that the effect is minor.

We are now in a better position to find the irreplaceable role of ECM stiffness and other characteristics in tumor progression. ECM stiffen used to be thought of as a disease consequence, but in fact, it was also a contributing factor to disease development. Integrated with the above research, we found that ECM stiffness can independently initiate EMT, or act as a costimulatory or “catalyst” in the tumor cell EMT process (**Figure** **5**). In general, matrix stiffness strengthens this process by mechanical transduction pathways under the action of EMT stimulating factors, including mainly the TGFβ family and others such as anoxia, oncogene, or defected environments. However, the effects of stiff and soft stroma on different types of cancer cells are not always the same. In most cases, the stiff substrate could promote EMT while soft substrate inhibits, but some studies have shown that the soft substrates cultivation of cells undergoes the EMT process more apparently,^[^ ^79^, ^80^, ^104^ ^]^ which suggests the complex effects of the ECM stiffness. Early studies mainly focused on breast, liver, and lung cancer and gradually involved other tumor fields in recent years, but the specific mechanism in diverse cancers has not yet been clarified. Furthermore, whether other stimuli in the TME, such as other cytokines or the stroma pH, participate in stiffness‐mediated EMT needs to be further demonstrated.

In addition to promoting the transformation of tumor cells into mesenchymal morphology, matrix stiffness has been found to be related to stem‐like cells in many studies and is related to the recurrence of drug resistance in cancer.^[^ ^136^, ^147^ ^]^ In addition to the interaction with tumor cells, ECM stiffness could also exert an influence on TAMs in TME,^[^ ^33^ ^]^ indicating the latent correlation between ECM stiffness and the immunoenvironment, which is associated with the immunotherapy response. Therefore, ECM stiffness, as a nonbiochemical factor, should not be neglected in tumor therapy. Currently, experiments targeting the ECM and mechanical transduction pathways are also underway.^[^ ^148^ ^]^ However, the complexity and pluripotency of ECM effects and mechanical transduction pathways greatly increase the difficulty of developing a safe and effective new drug. Moreover, compared with 2D culture, the cells cultured in 3D culture showed different cell reactions, which could also explain the in vivo insensitivity of drugs filtrated by non‐3D culture experiments. Therefore, for a step towards finding more effective cancer treatments, more research on the suitable culture systems and the mechanism of the role of the physical properties of the ECM in tumor progression has crucial implications.

**Figure 6:**
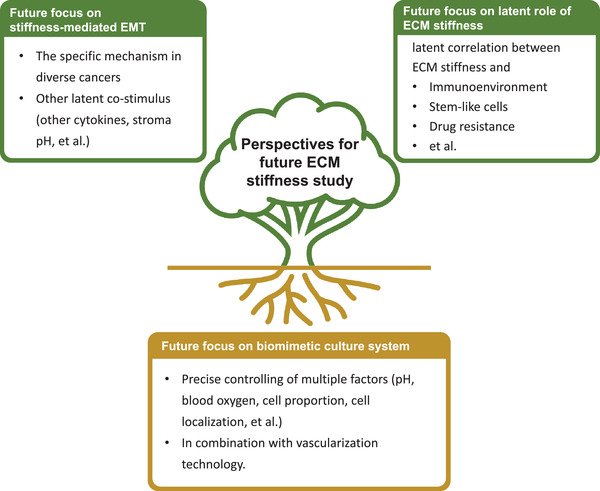
Perspectives for future ECM stiffness studies. Much work needs to be done, including identifying specific mechanisms in diverse cancers, deeply exploring the mechanism of ECM stiffness in tumor progression, and developing culture systems more akin to living tissues.

However, the current situation of the 3D system simulation is the only virtue of the material simulation, but the influence of multiple factors such as pH, blood oxygen environment, and cellular consortiums found in tumors should be considered. Thanks to the fast, flexible, and repeatable characteristics of 3D bioprinting, it is feasible to use 3D bioprinting to construct an in vitro patient‐specific cancer research platform that maximum simulates the tumor environment, with complete physical factors, precise control of cell proportion and localization, and vascularization. But the technological accessibility of 3D printing limits its wider application, which is also a problem that needs to be considered in the future (**Figure** **6**).

## Conflict of Interest

The authors declare no conflict of interest.

## Author Contributions

H.T., H.S., and J.Y. contributed equally to this work. J.R. and S.G. provided direction and guidance throughout the preparation of this manuscript. H.T. collected and interpreted studies and was a major contributor to the writing and editing of the manuscript. H.S. and J.Y. reviewed and made significant revisions to the manuscript. All authors read and approved the final manuscript.
